# Observation and Analysis of In Vitro Digestibility of Different Breads Using a Human Gastric Digestion Simulator

**DOI:** 10.3390/foods13203244

**Published:** 2024-10-12

**Authors:** Motomi Shibasaki, Tatsuro Maeda, Takayoshi Tanaka, Kenjiro Sugiyama, Hiroyuki Kozu, Ritsuna Noguchi, Takumi Umeda, Tetsuya Araki, Isao Kobayashi

**Affiliations:** 1 Faculty of Human Life, Jumonji University, 2-1-18 Sugasawa, Niiza 352-8510, Saitama, Japan; motomi48@jumonji-u.ac.jp; 2 Institute of Food Research, National Agriculture and Food Research Organization, 2-1-12 Kannondai, Tsukuba 305-8642, Ibaraki, Japan; kozuh095@affrc.go.jp (H.K.); umedat307@affrc.go.jp (T.U.); 3 Faculty of Health and Medical Science, Teikyo Heisei University, 2-51-4 Higashi-ikebukuro, Toshima Ward, Tokyo 170-8445, Japan; t.maeda@thu.ac.jp (T.M.); r.noguchi@thu.ac.jp (R.N.); 4 Graduate School of Agricultural and Life Sciences, The University of Tokyo, 1-1-1 Yayoi, Bunkyo Ward, Tokyo 113-8657, Japan; t-takayoshi@g.ecc.u-tokyo.ac.jp (T.T.); aaraki@g.ecc.u-tokyo.ac.jp (T.A.); 5 School of Advanced Engineering, Kogakuin University, 2665-1 Nakano-machi, Hachioji 192-0015, Tokyo, Japan; bt13171@ns.kogakuin.ac.jp

**Keywords:** bread, food bolus, in vitro digestion, gastric peristalsis, physical digestion, microstructure, gluten, starch

## Abstract

The digestion behavior of a food bolus comprising bread particles in the presence of gastric peristalsis remains poorly understood. This study systematically investigated the effect of bread type on in vitro gastric digestion behavior using a human gastric digestion simulator (GDS) that is capable of quantitatively simulating gastric peristalsis. A food bolus consisting of 60 g of bread (white bread, bagel, German bread, French bread, or croissant), 15 mL of a simulated salivary fluid, and 240 mL of a simulated gastric fluid was used for gastric digestion in vitro using the GDS for 3 h at 37 °C. Direct observation of the gastric digestion behavior in the GDS vessel demonstrated that the structure and composition of breads considerably influenced the physical digestion processes of bread particles. These processes include their fracture, rubbing, disintegration, swelling owing to the penetration of gastric fluid, and release of fat from their surface. Fluorescence microscopy enabled an improved understanding of the variations in the microstructure and major component distribution of the breads during the gastric digestion in vitro. The results showed how the different breads influenced gastric digestibility in vitro through quantitative gastric peristalsis. The GDS can also be applicable to studying gastric digestibility in vitro of other types of bread.

## 1. Introduction

Wheat is a major staple food grain worldwide and is produced and consumed in many parts of the world. The European Union countries produce and consume the largest amount of wheat, followed by China, India, Russia, and the USA [1]. Bread, which is made mainly from wheat flour, is a staple food in many countries. In Japan, bread consumption has increased since the 1970s, suggesting that bread, along with rice, is a staple food for the Japanese population. In 2011, the quantity of bread purchased by households with two or more members exceeded that of rice [2]. Presently, a wide variety of breads are available in Japan and other countries. For example, in addition to white bread, other breads are commonly consumed, such as croissants and brioches, which contain a lot of fat and oil, German bread and rice bread made from cereals other than wheat flour, and bagels and pretzels made by different fermentation and preparation methods.

Food digestion in humans involves oral digestion (chewing), swallowing, and gastric, small intestinal, and large intestinal digestion. These processes play a role in transforming food nutrients into absorbable states. Digestive processes, such as the grinding and rubbing of food particles, mixing of food particles and gastric fluid (pH 1–2), and decomposition of food components by digestive enzymes and gastric acid, take place in the human stomach [3]. Gastric content is transformed into a chyme state by physical and chemical digestion in the stomach, which is emptied from the pylorus into the duodenum.

The texture of bread is greatly affected by starch gelatinization and gluten formation during baking and the crumb and crust structures after baking. Therefore, the texture of bread affects mastication and digestion. Studies on the oral digestion of bread have shown that the type and internal structure of the main ingredients affect the amount of (simulated) absorbed salivary fluid [4,5,6,7]. Aleixandre et al. [7] investigated in vitro the oral digestion of white bread and bread rolls of the same composition made using wheat flour and reported that the structure of the crumb affects the particle size of the food bolus. In vitro studies on the gastric digestion of bread have also reported that the cohesive force of the food bolus decreases, and the disintegration rate increases with an increase in the amount of simulated salivary fluid that penetrates the interior of the bread [4].

Studies on the in vitro gastric digestion of foods suggest that the activity of the starch-digesting enzyme (α-amylase) contained in the food bolus influences gastric digestion [6]. However, previous studies have used a shaking test method that simulates chemical digestion, which does not adequately simulate the physical digestive behavior of the human stomach [4,5,6,7]. Therefore, the effects of different bread ingredients, structures, and additives on physical digestion in the stomach are largely unexplored.

Several methods have been adopted for investigating the gastric digestion of foods, including in vivo methods using magnetic resonance imaging and scintigraphy [8,9,10], in vitro static and shaking methods and digestion experiments [11], and in silico methods using computers [12,13]. In vivo methods face several hurdles in terms of ethical considerations and subject restrictions, whereas in silico methods are difficult to apply in complex food systems. In contrast, in vitro methods have fewer restrictions and can be used for conducting gastric digestion experiments on various types of foods. Conventional in vitro gastric digestion experiments are performed by simulating the chemical environment of the stomach (e.g., pH, enzymes, salt concentration, and temperature) in a glass or other container, regardless of the physical state of food (solid, semisolid, or liquid) [14]. Simulating the peristalsis that occurs in the human stomach wall is difficult using conventional methods; therefore, different types of digestion experimental equipment (e.g., TNO gastro-intestinal model (TIM), dynamic gastric model (DGM), human gastric simulator (HGS), and rope-driven in vitro human stomach model (RD-IV-HSM)) have been proposed to precisely examine the digestive behavior in the human stomach and small intestine [15,16,17]. A drawback of such equipment is that it is very complicated to use in the food industry.

The human gastric digestion simulator (GDS) has been developed based on the concept of simplifying the structure and function of the antrum, where the digestion of a food bolus actively occurs in the human stomach [18]. The GDS has a unique feature of directly observing the digestive behavior of food particles driven by the quantitative motion during peristalsis, which is not found in other equipment of gastric digestion. Starchy staple foods and protein foods have been used in studies using the GDS. Previous studies with the GDS have demonstrated that a reduction in particle size (fracture, rubbing, and disintegration) and swelling owing to the penetration of gastric fluid occur for some foods, which remarkably changes the tissue structure [18]. Therefore, the direct observation of intragastric digestion behavior may be useful for estimating the intragastric retention time, which could affect satiety. However, systematic studies on the intragastric digestive behavior of bread using the GDS are scanty.

This study aimed to systematically investigate and analyze the effects of different types of bread on gastric digestion behavior using the GDS. Specifically, we aimed to analyze the intragastric digestion behavior of a food bolus containing bread by direct observation and by analyzing the size distribution and microstructure of the gastric digesta by fluorescence microscopy.

## 2. Materials and Methods

### 2.1. Preparation of Bread Samples

In this study, five types of bread were selected (white bread, bagels, German bread, French bread, and croissants from the Nook Bread Shop (Kazo, Japan)) considering the differences in their ingredients and structure. Their main ingredients are cereal flour (wheat or rye flour), yeast, salt, and water. Simple breads without additional ingredients and those with many additional ingredients are called lean and rich breads, respectively. Among the breads selected for this study, white bread, bagels, German bread, and French bread were lean breads, and croissants are a rich bread (Table 1). The formulas of each bread are shown as the ratio of the ingredients to 100% wheat flour (or rye flour) (Table 2).

The bread particles tested were approximately 5 mm in size, which corresponds to the maximum size of a bread particle just before it is chewed and swallowed by a human. The particle size of a food bolus is affected by the hardness, length, and size of food, with a range <2 mm for hard foods, such as nuts, 4–5 mm for soft foods, and 0.6–6 mm for noodles such as pasta [19,20]. In this study, bread samples with particle sizes of approximately 5 mm were obtained by cutting bread into bite-sized pieces with a bread cutter mixer (Capsule Cutter Bonne, recolte, Winner’s Co., Ltd. (Tokyo, Japan)). Each bread sample weighed 60 g.

### 2.2. In Vitro Digestion Studies

#### 2.2.1. Preparation of Simulated Salivary and Gastric Fluids

The compositions of simulated salivary and gastric fluids were determined based on the compositions used in previous studies [21,22] (Table 3). The salts used in preparing the simulated digestive fluids were KCl, KH_2_PO_4_, NaHCO_3_, MgCl_2_(H_2_O)_6_, (NH_4_)_2_CO_3_, and CaCl_2_(H_2_O)_2_ (FUJIFILM Wako Pure Chemicals Co., Osaka, Japan). In addition, 51.0 U/mg α-amylase from Bacillus (#BCCB4170; Sigma-Aldrich, St. Louis, MO, USA) and 714 U/mg pepsin from porcine gastric mucosa (#BCBQ7633V; Sigma-Aldrich, Co., St. Louis, MO, USA) were used as digestive enzymes. Each simulated digestive fluid was heated to 37 °C in a constant-temperature water bath before the experiment. The pH of the simulated digestive fluids was adjusted to 1.3 using 6 M hydrochloric acid (FUJIFILM Wako Pure Chemicals Co., Osaka, Japan). For each digestion experiment, 15 mL simulated salivary fluid and 240 mL simulated gastric fluid were used.

#### 2.2.2. Gastric Digestion Using the GDS

A simulated food bolus was prepared by adding 15 mL of simulated salivary fluid to a cut bread sample (60 g), manually mixing it using a spoon at a constant speed (two times/s) for 90 s, and allowing the sample to stand for 30 s.

**Figure 1:**
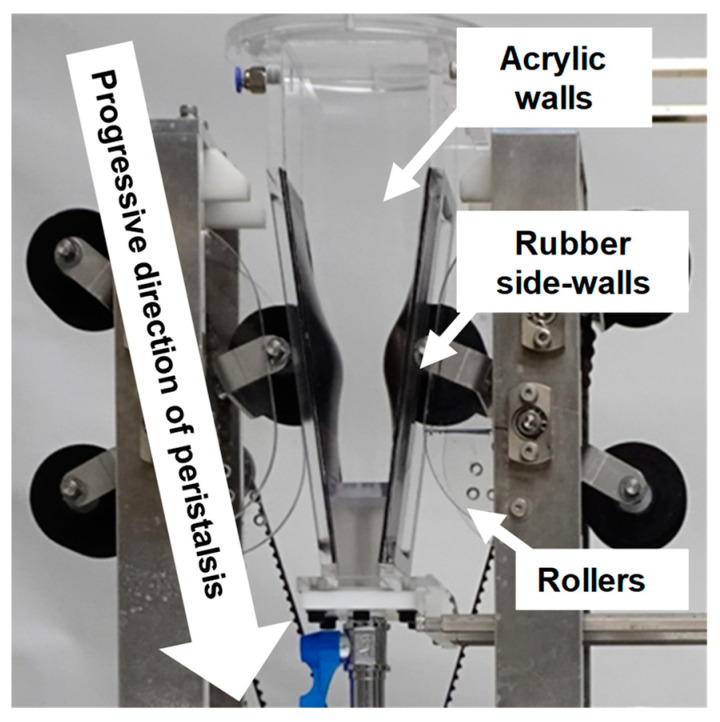
Key components of the GDS.

In vitro gastric digestion was performed at 37 °C for 180 min using the GDS (Figure 1) [22]. The key components of the GDS (Figure 1) consist of a 550 mL vessel simulating the antrum, rollers capable of generating peristaltic waves, and a temperature control unit. Rubber side-walls of the vessel simulate the stomach wall, and transparent, flat acrylic walls of the vessel allow direct observation of the digestion behavior of the gastric contents. The top of the vessel is also open to allow sampling and pH measurement of the gastric contents. The speed and cycle of gastric peristalsis were set to 2.5 mm/sec and 1.5 cycles/min, respectively. pH was measured every 30 min using an ISFET electrode (LAQUAact D-72F; Horiba, Ltd., Kyoto, Japan), and solid fractions of the gastric contents were collected. pH was measured using an electrode positioned 10 mm below the liquid surface of the gastric contents. A video camera (WZX992M; Panasonic Corporation, Tokyo, Japan) was used to directly observe and record the digestion process.

After the digestion experiment, the gastric digesta sample was classified using sieves with different openings (d: 2.00, 0.60, and 0.10 mm). The size and morphology of the particles blocked by each sieve were observed, and wet weights were measured. The wet weights for each size fraction were the average of the data obtained from two gastric digestion experiments for each bread sample. The dry weights for each size fraction were measured using samples dried in a vacuum oven (AVO-250NS, As One Co., Ltd., Osaka, Japan) at 105 °C for 12 h.

### 2.3. Fluorescence Microscopic Analysis

The microstructural changes in various breads before and after the GDS digestion experiment were observed using a fluorescence microscope (BX53, Olympus Corporation, Tokyo, Japan). The solid fractions of gastric contents collected immediately after digestion were rough-cut and filled into plastic cells with an optimal cutting temperature compound. The plastic cells were quickly frozen at −80 °C. The frozen contents were cut to 20 μm slices using a cryostat microtome (CM1100; Leica, Wetzlar, Germany) and fixed onto glass slides. The bread samples were stained with 0.5% (*w*/*w*) solutions of acid magenta for staining times of 5 min. The staining process was performed at ambient temperature. After staining, the samples were washed with water and air-dried on microscope glasses. The stained samples were observed through a fluorescent microscope with the following filter settings: WU (blue), excitation wavelengths in the range of 330–385 nm, and observation wavelengths in the range longer than 420 nm. An eyepiece of 10× and objective lenses of 4×, 10×, or 20× were combined for observation at 40×, 100×, or 200× magnification, respectively. Fluorescent images were captured using a CCD camera (DP74; Olympus Corporation, Tokyo, Japan). The exposure time was set between 10 and 100 ms, and the film speed was set to ISO1600. Three evaluation axes (coarseness of the gluten network, dispersion of gluten and starch, and size and distribution of the bubbles) were generated for evaluating the acquired images [23].

## 3. Results and Discussion

### 3.1. Direct Observation of Digestion of Different Breads in the GDS

**Figure 2:**
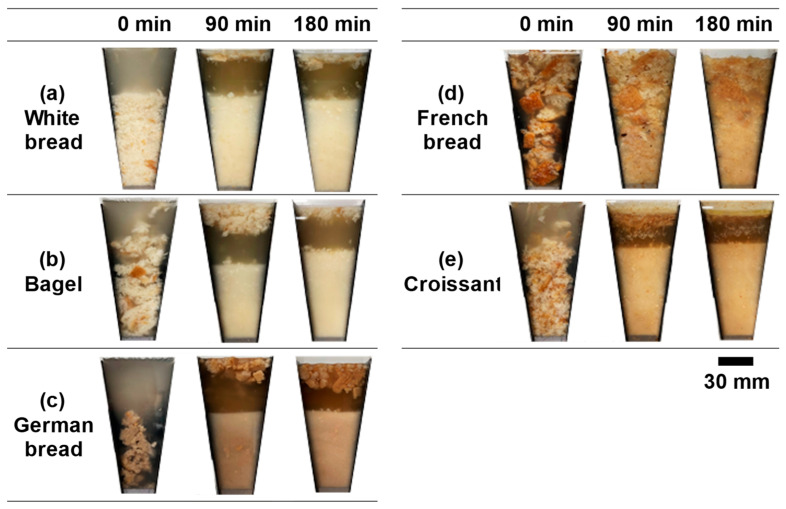
Images of the different breads during the GDS experiments.

Figure 2 shows the results of observations of the gastric contents containing bread during in vitro digestion using the GDS. After the start of digestion, the rollers contacted the vessel wall, and the resulting peristaltic motion progressed toward the bottom of the vessel. Progressive gastric peristalsis caused compression and mixing of the gastric contents. Regardless of the bread type, the gastric contents consisted of two layers: a liquid layer at the top and a particle sediment layer at the bottom. For some breads, a white liquid layer appeared on the upper surface of the particle sediment layer as gastric digestion progressed.

The porous structure of white bread, which consists of meshes and large voids, makes the dough soft and the structure easily deformable. Immediately after the start of gastric digestion, the simulated gastric fluid penetrated the interstitial spaces of the food bolus and interior of the white bread, causing it to swell (Figure 2a). Subsequently, the food bolus particles were rapidly dispersed into the simulated gastric fluid via peristalsis. After 90 min, most fine particles sedimented in the particle layer, whereas a small number of coarse particles were observed on the upper surface of the liquid layer. Similar behaviors have been observed for a commercialized white bread [24], where the food bolus particles quickly dispersed in the simulated gastric fluid via peristalsis and became fine as the digestion time passed.

Bagels have a dense porous structure with a high density of voids in the crumb. Immediately after the start of gastric digestion, some of the food boluses broke into coarse particles and floated in the simulated gastric fluid (Figure 2b). Subsequently, the simulated gastric fluid penetrated the tiny spaces between the coarse particles, thereby forming a layer of coarse particles near the top of the liquid layer, a layer of fine particles near the bottom, and a layer of slightly coarse particles in the middle. After 90 min, a coarse particle layer was observed on the upper surface of the liquid layer, and a particle sediment layer consisting of fine and slightly coarse particles was noticed. At the end of experiment, the volume of the particle sediment layer increased further, and the fine particles formed a chyme-like state.

Since the main ingredient of German bread is rye flour, it has a dark reddish-brown color and high nutritional value owing to its richness in dietary fibers such as β-glucan. In addition, German bread has a moist texture owing to its high content of soluble dietary fibers and ability to absorb moisture. In contrast, German bread has low elasticity and a dense porous structure owing to low glutenin and high gliadin contents, which contribute to the formation of gluten. Therefore, the German bread became sticky when mixed with the simulated salivary fluid, forming a food bolus faster than the other breads. Furthermore, the food bolus was composed of coarse particles with a high crust content and fine particles with a high crumb content. The German bread maintained a dumpling-like food bolus immediately after the start of digestion (Figure 2c), but it broke up into coarse and fine particles within approximately 5 min by gastric peristalsis. Consequently, a layer of coarse particles on the upper surface and a layer of fine particles at the bottom of the liquid layer were formed. Even after the simulated gastric fluid gradually penetrated the coarse particles, which had a high proportion of hard and firm crusts, they floated in a swollen state. The sediment layer consisting of fine particles formed a chyme-like state in approximately 90 min and partially and temporarily floated by gastric peristalsis. At this time, the simulated gastric fluid in the GDS vessel had a dark reddish-brown color. At the end of the experiment, the volume of the coarse particle layer on the upper surface of the liquid layer slightly increased, and the stickiness and chyme formation in the particle sediment layer progressed.

French bread with an elongated shape is made by baking dough with slits on its surface; it consists of a hard crust and a crumb of a coarse and porous structure with irregular voids of various sizes. Immediately after the start of digestion, the simulated gastric fluid quickly penetrated the crumb, resulting in the swelling and floating of food bolus particles throughout the gastric contents (Figure 2d). After 30 min, the food bolus became noticeably fine via gastric peristalsis. After 90 min, particle disintegration and chyme formation progressed in the lower half of the gastric contents. The coarse particles in the upper part of the gastric contents became relatively fine and maintained their swollen state and shape in the simulated gastric fluid. Unlike the digestive behavior of other breads, French bread was present throughout the gastric contents immediately after the start of digestion, and its volume increased because of the swelling of the food bolus. By the end of the experiment, further particle disintegration led to the formation of gastric contents consisting of an upper layer rich in crusts and a lower layer rich in crumbs.

A croissant is a multilayered bread made of dough and fat through baking and is rolled repeatedly to form a thin, multilayered structure of fat and oil. The layers are expanded by vapor pressure during baking. Immediately after the start of digestion, the food bolus with a multilayered structure moved upward and downward by gastric peristalsis while floating in the simulated gastric fluid (Figure 2e). After 30 min, large flakes of the food bolus floated on the surface of the liquid layer, and fine particles settled in the bottom sediment layer. After 90 min, the food bolus at the top of the liquid layer settled, and the volume of the particle sediment layer increased as the food bolus became fine via gastric peristalsis. Additionally, a fat layer appeared below the particle layer on the upper surface of the liquid layer, probably owing to the higher fat content of croissants compared to that of the other breads used in this study. Although the fat layer remained until the end of the experiment, its volume did not increase. This suggests that the fat contained in the croissant was released into the simulated gastric fluid within approximately 90 min.

Because the surface and internal structures of breads depend on their ingredients and preparation method, the percentage of crust after baking affects the force and number of mastications and the amount of impregnated salivary fluid [25]. The degree of disintegration and behavior of the crust and crumb of each bread were found to be different. The crusts were difficult to disintegrate, and the penetration of simulated gastric fluid was relatively slow. The components that impart browning to the crust were released into the simulated gastric fluid during the experiment. In contrast, the crumb was composed of a reticular skeleton and voids; thereby, rapid penetration of the simulated gastric fluid into the crumb and the disintegration of the crumb particles by gastric peristalsis can occur. In particular, the size difference between coarse and fine particles was large in the French bread, owing to the high proportion of crust and the large variation in the void size of crumbs. These results suggest that French bread swells upon the penetration of simulated gastric fluid and maintains its swollen state, which may lead to prolonged gastric retention time and increased satiety. Differences in the ingredients, composition, and structure of breads can also affect the gastric digestion behavior.

### 3.2. Particle Size Distribution of the Gastric Digesta

**Figure 3:**
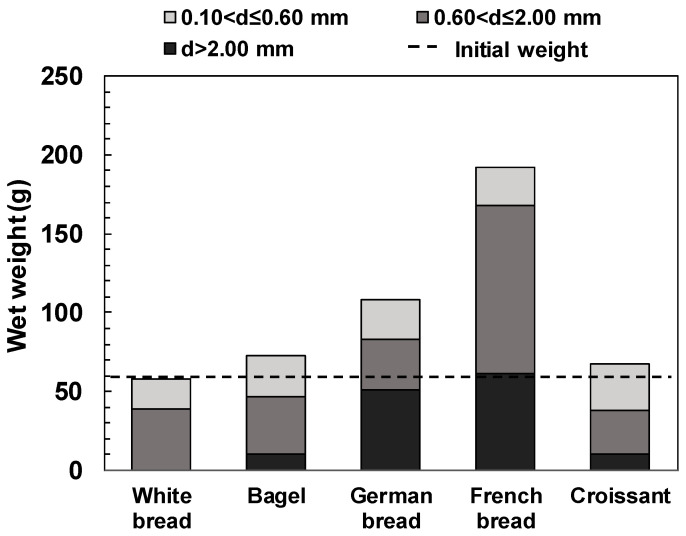
The wet weight of each size fraction regarding the classified gastric digesta of different breads.

**Figure 4:**
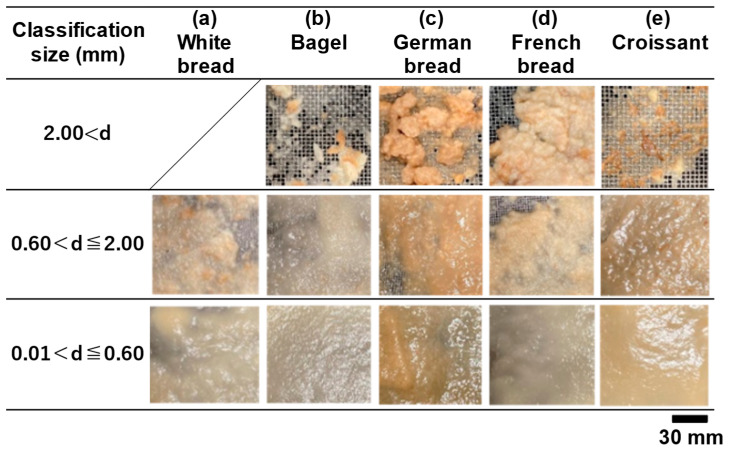
Images of the classified gastric digesta of different breads.

Figure 3 shows the wet-weight distribution of the gastric digesta remaining in each sieve. The size of the pylorus, which is the exit point of the human stomach, is approximately 2 mm, and particles smaller than the pylorus are emptied into the duodenum. Figure 4 shows images of the gastric digesta classified after the digestion experiment.

The total wet weight of each gastric digesta remaining on the sieve was 58.0 g for the white bread, 72.6 g for the bagel, 108.5 g for the German bread, 253.5 g for the French bread, and 67.9 g for the croissant. The shape, color, and gloss of particles remaining on the sieve varied depending on the degree of disintegration of bread particles. The wet weights of the gastric digesta remaining on the 2.00 mm sieve were 10.2 g for the bagel, 50.9 g for the German bread, 122.3 g for the French bread, and 10.8 g for the croissant. In the case of the French bread, this fraction accounted for approximately 60% of the total wet weight because the coarse particles were difficult to disintegrate. The gastric digesta of the white bread did not remain on the 2.00 mm sieve. The gastric digesta remaining on the 0.60 mm sieve contained a mixture of crust and crumb, regardless of the bread type and particles that affected browning of the crust.

In the case of the white bread, we considered that the simulated gastric fluid promoted particle disintegration by permeation into the interstitial spaces inside crumbs, quickly progressing to form a chyme-like state (Figure 4a). In the bagel, the dense crumb structure inhibited the entry of enzymes in the simulated gastric fluid into the crumb [26]. This phenomenon may have caused the disintegration of food bolus particles by gastric peristalsis (Figure 4b). As for the gastric digesta including German bread, coarse particles of hard crusts maintained intact shape and size (Figure 4c). The gastric digesta of the German bread, which is mainly composed of rye, was somewhat shiny and sticky. This indicated that diffusion of this viscous material made the particle surfaces slippery, thereby inhibiting their disintegration. For the gastric digesta containing French bread, the penetration of the simulated gastric fluid resulted in the formation of swollen and coarse particles (Figure 4d), while particle size reduction was consistent with suppressed intragastric digestion behavior. These behaviors differed from those of other breads, which might affect the release and diffusion of nutrients. Regarding the gastric contents of the croissant, we observed that the crumbs became relatively thin and fine (Figure 4e). Because croissants are rich in fat, the chyme-like digesta was very shiny and had a smooth and paste-like appearance compared to that of the other breads.

The dry weight of the gastric digesta containing bread was nearly equal to that of the bread sample itself (Figure S1). The white bread and bagel had relatively high dry weights of 0.60 mm or smaller fractions owing to the absence or low amount of 2.00 mm or larger fractions. In contrast, the German and French breads had a high number of fractions with 0.06–2.00 mm size. The dry weights of the fractions were 23.9 and 35.6 g for the German and French breads, accounting for 53.3% and 74.0% of their total dry weights, respectively.

The above results indicate that breads with a hard crust or viscous food bolus, such as German and French breads, are difficult to disintegrate. This digestion behavior differs from that of the other breads, suggesting that ingestion of these two types of bread may lead to a prolonged gastric retention time.

### 3.3. Variation in pH of the Gastric Contents during Digestion

Figure S2 shows the variation in pH of the gastric contents during the digestion of different breads. The range of increase in the pH of the gastric contents during digestion was 0.9 for the white bread, 0.3 for the bagel, 0.4 for the German bread, 0.7 for the French bread, and 0.9 for the croissant. The white bread and croissant had the highest increases in pH. White bread has a crumb with a thin-walled network structure and high water content. The simulated gastric fluid quickly penetrated the crumb, and the food bolus rapidly disintegrated by gastric peristalsis. This may have led to the dissolution and diffusion of the soluble proteins of the white bread, resulting in a relatively high increase in pH. The high fat content of the croissant may have affected the pH increment after 150 min. The time required for the disintegration of bolus particles and formation of the chyme-like state depended on the bread type, suggesting that the nutrients released from bolus particles affect the change in intragastric pH. The maximum pH of the gastric contents at the end of experiment was 2.39, which was lower than the pH (~3) for inactivating pepsin [27].

### 3.4. The Microstructure of Gastric Digesta Observed by Fluorescence Microscopy

**Figure 5:**
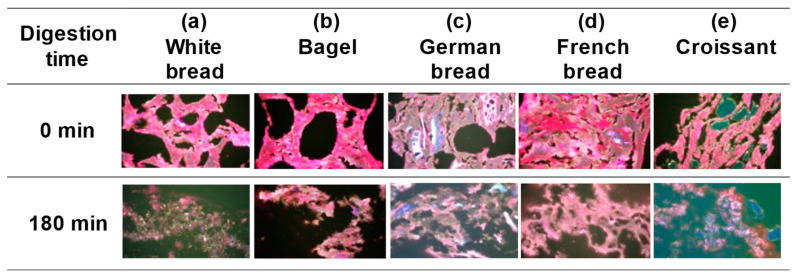
Fluorescent micrographs of the gastric content and digesta of different breads. Red, white, translucent, black, and green denote gluten, ungelatinized starch granules, gelatinized starch granules, bubbles, and oil droplets, respectively.

Gluten is a major component of the dough of cereal flour products and influences food texture and flavor. The network structure of gluten and the matrix of starch granules determine the texture of bread [23]. The structure of gluten in bread dough is typically observed using optical microscopy (bright-field, polarized light, and fluorescence microscopy), confocal laser microscopy, and excitation fluorescence imaging. Optical microscopy using fluorescent staining can clearly distinguish gluten from starch granules by combining a specific staining agent and fluorescence wavelength. Maeda et al. [23] used fluorescence microscopy for visualizing the gluten structure and starch distribution state of different types of wheat flour products and evaluating their characteristics. Figure 5 shows the microstructures of fluorescently labelled bread samples and their gastric digesta.

In the white bread, a thin-walled network structure composed mainly of gluten was formed at the beginning of digestion, and voids of non-uniform size were dispersed in the network structure (Figure 5a). Starch granules were also dispersed within the network structure. Most of the starch granules were gelatinized; however, ungelatinized starch granules were also observed. By the end of experiment, most of the gluten had been decomposed by pepsin, and fine starch granules remained dispersed.

At the beginning of experiment, the bagel had a dense and thick-walled network structure composed mainly of gluten, with slightly smaller and more uniformly sized voids than those of the white bread (Figure 5b). In addition to the high fraction of gelatinized starch granules, a matrix of gelatinized starch granules and gluten was tightly bound together. At the end of the experiment, some gluten remained inside the network structure, whereas the gluten near the wall surface was decomposed, and starch granules were disintegrated.

In the German bread, a very thick-walled network structure was observed at the beginning of the experiment (Figure 5c). Inside the thick walls, a matrix was formed, in which gluten surrounded gelatinized starch granules, which may have contributed to the crumb strength. At the end of the experiment, a network structure consisting of partially decomposed gluten and surrounding starch granules was retained.

In the French bread, at the beginning of experiment, a network structure with various wall thickness was formed, which was mainly composed of gluten, and starch granules were contained inside the overcrowded walls (Figure 5d). As shown in Figure 4d, the interior of the mesh contained numerous microvoids. These results suggest that French bread has a microstructure different from that of the other breads. At the end of the experiment, a network structure composed of partially decomposed gluten and gelatinized starch was observed.

The microstructure of the croissant had characteristics that were not observed in the other breads. At the beginning of the experiment, a laminated structure was formed with oil droplets sandwiched between gluten-based walls (Figure 5e). Uneven and elongated voids were also observed between the adjacent layers. At the end of the experiment, the stacked structure disintegrated and disappeared, whereas many ungelatinized starch granules remained.

Microscopic observation and analysis of the fluorescently labelled gastric contents and digesta of different breads revealed that their microstructure, the thickness and distribution of gluten-based walls, and the size, shape, and distribution of the voids pronouncedly varied depending on the bread type. The microstructures of gluten and starch granules depended on bread ingredients and cooking methods, suggesting that these factors affect gastric digestion, including gluten decomposition.

The nutritional composition of each bread is shown in Table S1 considering the ratio of the ingredients to 100% wheat flour (or rye flour). We assumed that the network structure of different breads is influenced by their preparation method and secondary ingredients. Gelatinization and retrogradation of the starch were observed in different breads, whereas the bubble walls and network structure were maintained. The protein content of the breads may also influence the thickness and coarseness of the bubble walls.

The results obtained in this study indicate the applicability of the GDS to other types of bread.

## 4. Conclusions

We evaluated the in vitro gastric digestibility of five different breads using the GDS. The results demonstrate that the surface and internal structures and composition of the breads pronouncedly affected the physical gastric digestion processes, including the fracture, rubbing, and disintegration of bread particles, swelling due to the penetration of the gastric fluid, and the release of fat from the particle surface. For the white bread, bagel, German bread, and croissant, the bread particles gradually became smaller, which is mainly attributable to their fracture, rubbing, and disintegration. In contrast, for the French bread with a coarse porous internal structure, remarkable swelling of bread particles, in addition to particle disintegration, was observed, indicating the possibility of prolonged gastric retention time in the stomach. Observations using fluorescent microscopy showed the difference in the microstructure of the breads and the distribution of gluten and starch in the walls. The fluorescent micrographs of the gastric digesta also demonstrated how the microstructure and major component distribution of different breads varied during the in vitro gastric digestion experiments. These findings provide useful insights into in-depth understanding of the gastric digestibility of breads, which may help in preparing novel bread products with improved design for efficient gastric digestion. The GDS can also be applied to study gastric digestibility in vitro of other types of bread (e.g., rice flour breads and rice flour blend breads).

## Figures and Tables

**Table 1:** Categories and major features of the bread used in this study.

	Lean Breads	Rich Breads
Types	White bread, bagel, German bread, French bread, etc.	Croissant, Danish, Brioche, etc.
Major features	Simple bread made by mixing flour, yeast, salt, and water, without secondary ingredients.	Bread made by mixing/combining flour, yeast, salt, and water with secondary ingredients such as butter, sugar, eggs, and dairy products.

**Table 2:** Formulas of the bread used in this study.

	White Bread	Bagel	German Bread	French Bread	Croissant
Wheat flour	100	100	30	100	100
Rye flour	–	–	70	–	–
Salt *	2	2	2	2.1	2.1
Yeast *	12	2.5	2.8	5.7	12.3
Water or milk *	70	55	73	73	52
Sugar *	6	7	–	–	15
Butter (unsalted) *	7	–	–	–	50

* The percentage (%) of each ingredient in relation to the weight of wheat and rye flour.

**Table 3:** Composition of simulated salivary and gastric fluids *.

Simulated Salivary Fluid			Simulated Gastric Fluid		
KCl	1.126	g/L	KCl	0.514	g/L
KH_2_PO_4_	0.503	g/L	KH_2_PO_4_	0.122	g/L
NaHCO_3_	1.142	g/L	NaHCO_3_	2.1	g/L
MgCl_2_(H_2_O)_6_	0.03	g/L	NaCl	2.76	g/L
(NH_4_)_2_CO_3_	0.006	g/L	MgCl_2_(H_2_O)_6_	0.02	g/L
CaCl_2_(H_2_O)_2_	0.221	g/L	(NH_4_)_2_CO_3_	0.074	g/L
α-Amylase	150	U/mL	CaCl_2_(H_2_O)_2_	0.022	g/L
HCl	1.1	mM	Pepsin	4000	U/mL
			HCl	49.8	mM
pH		6.8	pH		1.3

* Reference [22].

## Data Availability

The original contributions presented in the study are included in the article/Supplementary Material, further inquiries can be directed to the corresponding author.

## Supplementary Materials

The following supporting information can be downloaded at https://www.mdpi.com/article/10.3390/foods13203244/s1, Figure S1. The dry weights of each size fraction regarding the classified gastric digesta of different breads. Figure S2. Variations in pH of the gastric content of different breads as a function of digestion time. Table S1. Nutritional composition of the breads used in this study.
