# Supplementary material for: Observation and Analysis of In Vitro Digestibility of Different Breads Using a Human Gastric Digestion Simulator
Source: Foods. 2024 Oct 12;13(20):3244. doi: 10.3390/foods13203244 (PMC11507474; doi:10.3390/foods13203244)
Supplement: Supplementary file 1 [file foods-13-03244-s001.zip › foods-3213261-supplementary.pdf]

Supplementary Materials

Observation and Analysis of In Vitro Digestibility of Different Breads Using a Human Gastric Digestion Simulator

Motomi Shibasaki [1,2], Tatsuro Maeda [3], Takayoshi Tanaka [4], Kenjiro Sugiyama [5], Hiroyuki Kozu [2], Ritsuna Noguchi [3], Takumi Umeda [2], Tetsuya Araki [4] and Isao Kobayashi [2,*]

[1] Faculty of Human Life, Jumonji University, 2-1-18 Sugasawa, Niiza 352-8510, Saitama, Japan
[2] Institute of Food Research, National Agriculture and Food Research Organization, 2-1-12 Kannondai, Tsukuba 305-8642, Ibaraki, Japan
[3] Faculty of Health and Medical Science, Teikyo Heisei University, 2-51-4 Higashi-ikebukuro, Toshima Ward, Tokyo 170-8445, Japan
[4] Graduate School of Agricultural and Life Sciences, The University of Tokyo, 1-1-1 Yayoi, Bunkyo Ward, Tokyo 113-8657, Japan
[5] School of Advanced Engineering, Kogakuin University, 2665-1 Nakano-machi, Hachioji 192-0015, Tokyo, Japan
* Correspondence: isaok@affrc.go.jp; Tel.: +81-29-838-8026

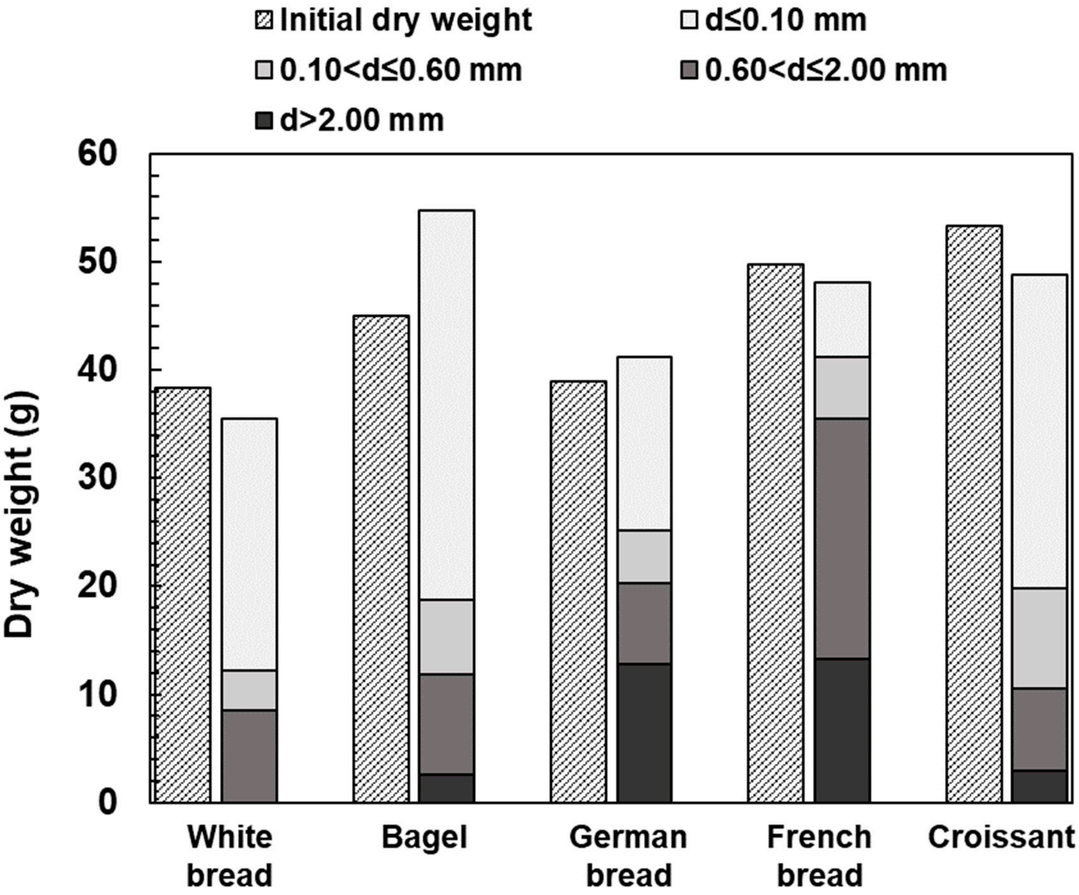

Figure S1. The dry weights of each size fraction regarding the classified gastric digesta of different breads.

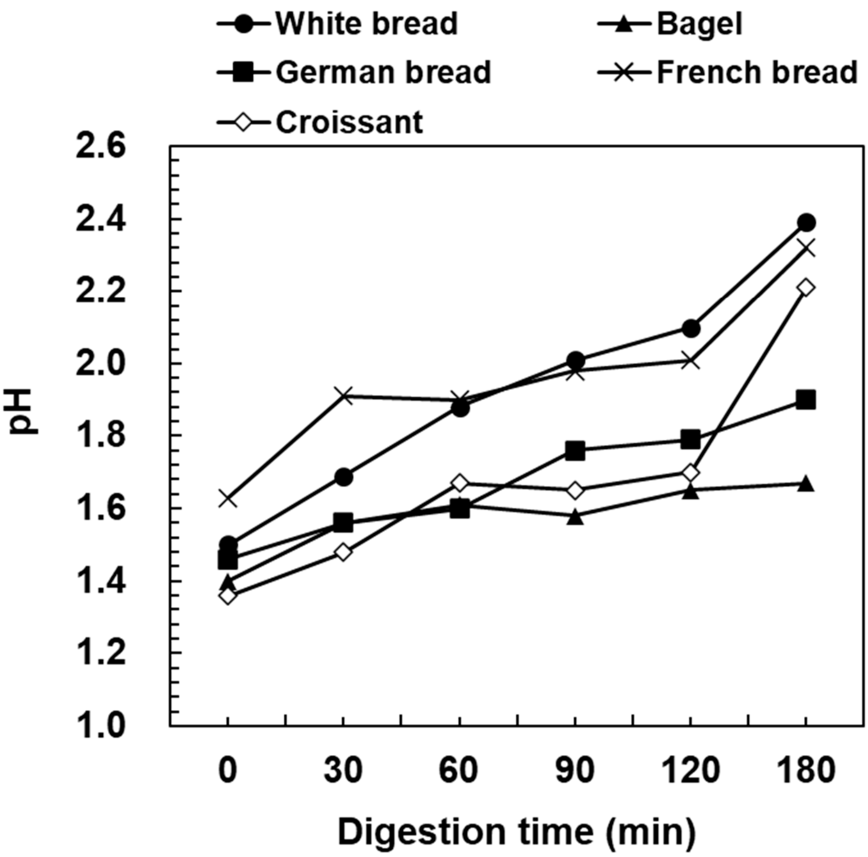

Figure S2. Variations of pH of the gastric content of different breads as a function of digestion time.

Table S1. Nutritional composition of the breads used in this study.*

Nutrition Facts	White bread **	Bagel	German bread	French bread	Croissant **
**Energy (kcal)**	466	372	336	354	801
**Protein (g)**	16.1	12.7	10.5	13.9	15.8
**Fat (g)**	10.2	1.7	1.8	1.9	45.2
**Carbohydrate (g)**	82.5	79.8	75.8	74.2	90.8
**Starch (g)**	66.6	66.5	60.4	66.6	66.6
**Dietary fiber (g)**	3.9	3.5	10.8	4.6	4.0

* Formula of the breads are presented in Table 2.

** Natural yeast was used. Dry yeast was used for the other breads.
